# Women's and care providers' perspectives of quality prenatal care: a qualitative descriptive study

**DOI:** 10.1186/1471-2393-12-29

**Published:** 2012-04-13

**Authors:** Wendy Sword, Maureen I Heaman, Sandy Brooks, Suzanne Tough, Patricia A Janssen, David Young, Dawn Kingston, Michael E Helewa, Noori Akhtar-Danesh, Eileen Hutton

**Affiliations:** 1 School of Nursing and Department of Clinical Epidemiology and Biostatistics, Faculty of Health Sciences, McMaster University, 1280 Main Street West, Hamilton, Ontario L8S 4K1, Canada; 2 Faculty of Nursing, University of Manitoba, 89 Curry Place, Winnipeg, Manitoba R3T 2N2, Canada; 3 School of Nursing, Faculty of Health Sciences, McMaster University, 1200 Main Street West, Hamilton, Ontario L8N 3Z5, Canada; 4 Department of Paediatrics and Community Health Sciences, Faculty of Medicine, University of Calgary and Alberta Centre for Child, Family and Community Research, 2888 Shaganappi Trail NW, Calgary, Alberta T3B 6A8, Canada; 5 School of Population and Public Health, University of British Columbia, 2206 East Mall, Vancouver, British Columbia V6T 3Z1, Canada; 6 IWK Health Centre and Department of Obstetrics and Gynecology, Faculty of Medicine, Dalhousie University, 5980 University Avenue, P.O. Box 9700, Halifax, Nova Scotia B3K 6R8, Canada; 7 Faculty of Nursing, University of Alberta, 5-258 Edmonton Clinic Health Academy, 11405-87th Avenue, Edmonton, Alberta T6G 1C9, Canada; 8 St. Boniface General Hospital and Department of Obstetrics, Gynecology and Reproductive Sciences, Faculty of Medicine, University of Manitoba, 409 Tache Avenue, Winnipeg, Manitoba R2H 2A6, Canada; 9 School of Nursing and Department of Clinical Epidemiology and Biostatistics, Faculty of Health Sciences, McMaster University, 1280 Main Street West, Hamilton, Ontario L8S 4K1, Canada; 10 Department of Obstetrics and Gynecology and Department of Clinical Epidemiology and Biostatistics, Faculty of Health Sciences, McMaster University, 1280 Main Street West, Hamilton, Ontario L8S 4K1, Canada

## Abstract

**Background:**

Much attention has been given to the adequacy of prenatal care use in promoting healthy outcomes for women and their infants. Adequacy of use takes into account the timing of initiation of prenatal care and the number of visits. However, there is emerging evidence that the quality of prenatal care may be more important than adequacy of use. The purpose of our study was to explore women's and care providers' perspectives of quality prenatal care to inform the development of items for a new instrument, the Quality of Prenatal Care Questionnaire. We report on the derivation of themes resulting from this first step of questionnaire development.

**Methods:**

A qualitative descriptive approach was used. Semi-structured interviews were conducted with 40 pregnant women and 40 prenatal care providers recruited from five urban centres across Canada. Data were analyzed using inductive open and then pattern coding. The final step of analysis used a deductive approach to assign the emergent themes to broader categories reflective of the study's conceptual framework.

**Results:**

The three main categories informed by Donabedian's model of quality health care were structure of care, clinical care processes, and interpersonal care processes. Structure of care themes included access, physical setting, and staff and care provider characteristics. Themes under clinical care processes were health promotion and illness prevention, screening and assessment, information sharing, continuity of care, non-medicalization of pregnancy, and women-centredness. Interpersonal care processes themes were respectful attitude, emotional support, approachable interaction style, and taking time. A recurrent theme woven throughout the data reflected the importance of a meaningful relationship between a woman and her prenatal care provider that was characterized by trust.

**Conclusions:**

While certain aspects of structure of care were identified as being key dimensions of quality prenatal care, clinical and interpersonal care processes emerged as being most essential to quality care. These processes are important as they have a role in mitigating adverse outcomes, promoting involvement of women in their own care, and keeping women engaged in care. The findings suggest key considerations for the planning, delivery, and evaluation of prenatal care. Most notably, care should be woman-centred and embrace shared decision making as an essential element.

## Background

Prenatal care has become one of the most widely used preventive health care services in developed countries [1,2]. Broadly defined, it encompasses "the detection, treatment, or prevention of adverse maternal, fetal, and infant outcomes as well as interventions to address psychosocial stress, detrimental health behaviors such as substance abuse, and adverse socioeconomic conditions" (p.116) [1]. Much attention has been given to the adequacy of prenatal care use in mitigating poor outcomes for women and their infants. Adequacy of utilization has been conceptualized as consisting of two dimensions: the timing of initiation of prenatal care and the number of prenatal visits, taking gestational age at entry into care and at delivery into consideration [3]. As noted by Kotelchuck [3], adequacy does not take into account the content or quality of care that is delivered but rather focuses only on quantifying its use.

There is emerging evidence that the quality of prenatal care, i.e., what is actually done during the giving and receiving of care, may be more important than the quantity of care. Ricketts, Murray, and Schwalberg [4], for instance, found that providing enhanced prenatal care to high-risk women that specifically addressed lifestyle and psychosocial characteristics was effective in resolving risk factors and, subsequently, low birth weight risk. In another study an association was found between the health promotion content of prenatal care received at a low-risk clinic and healthy behaviours in pregnancy, including reduced substance use [5]. Evaluations of Centering Pregnancy^©^, a group model of prenatal care that allows more time with care providers than traditional care and is relationship-centered, suggest its potential effectiveness in reducing negative birth outcomes [6]. A randomized controlled trial of Centering Pregnancy^© ^demonstrated improvements in gestational age, maternal psychosocial function, breastfeeding initiation, and satisfaction with care [7].

In light of this evidence that suggests the importance of quality of care and evidence that reducing the frequency of prenatal visits for low-risk healthy women does not adversely affect maternal or neonatal outcomes, the need for the usual 14 to 16 visits recommended by some professional organizations has been questioned [8]. In fact, a recommended schedule of fewer visits for such women was proposed over 20 years ago by an expert panel of the U.S Public Health Service's Low Birth Weight Prevention Work Group [9]. This recommendation was based on the assumption that high quality care is offered [10].

There is no agreement, however, as to what constitutes quality prenatal care. The list of nine indicators of quality prenatal care developed by a working group of the Royal College of Obstetricians and Gynaecologists reflect very defined medical aspects of care (e.g., Rhesus antibody screening, detection of and use of external cephalic version for breech presentation, steroid administration in preterm delivery) [11]. Adherence to evidence-based clinical practice guidelines that are both applicable to the population of childbearing women and to midwifery practice has been suggested as a strategy to maintain quality in antenatal care delivered by midwives [12]. Kirkham, Harris, and Grzybowski [13] similarly proposed that prenatal care should be based on "the best available evidence" but added that this evidence should be integrated "into a model of informed, shared decision making" (p. 1307). While noting that medical procedures are important, Alexander and Kotelchuck [1] suggested that parameters for assessing quality of prenatal care should take into account the provision of health education, assessment of the need for and referral to ancillary services (e.g., nutrition support, social services), and the nature of patient-provider-system interactions.

Given the wide variation in opinions about the essential elements of quality prenatal care, the inconsistency in approaches to assessing quality of prenatal care in the published literature is not surprising. Research in this area has largely been atheoretical, few studies have considered women's perspectives, and much of the focus has been on medical or clinical aspects of care to the exclusion of interpersonal processes. Moreover, studies seeking to examine the relationships between quality of prenatal care and perinatal outcomes have been hindered by the lack of a theoretically-grounded and psychometrically-tested instrument. To fill this gap we conducted research with the aim of developing and testing an instrument to measure quality of prenatal care, the Quality of Prenatal Care Questionnaire. As a first step in instrument development, semi-structured interviews were conducted with women and prenatal care providers to ascertain their views of quality care. Understanding what patients value is particularly critical in a prenatal care context as engagement of women in care is important for early initiation and continuation of care over a relatively short time period for health promotion, prevention of adverse outcomes, and early identification of and intervention for health risks [14]. Additionally, there is evidence that engagement in prenatal care is predictive of future use of preventive health services, including well-child care [15].

The purpose of this article is to describe women's and prenatal care providers' perspectives of quality prenatal care. In doing so, the research adds to our understanding of specific dimensions of prenatal care that ultimately might contribute to healthy outcomes for women and their infant. We received ethics approval for this study from Hamilton Health Sciences/McMaster University Faculty of Health Sciences Research Ethics Board and the ethics committee responsible for the conduct of research at each participating site.

## Methods

A qualitative descriptive exploratory design was used to understand women's and care providers' perspectives of quality prenatal care. As noted by Sandelowski [16], qualitative description is especially useful in obtaining straight descriptive answers to questions of special relevance to practitioners and policy makers. The conceptual framework that guided the study was derived from Donabedian's [17] systems-based model of quality health care. It encompasses three aspects of care: structure, processes, and outcomes. Structure refers to attributes of the setting in which health care is delivered and received; the domains of care structure include physical setting and staff characteristics [17,18]. Process of care refers to what is actually done in the delivery and receipt of care and incorporates two key components: clinical care and interpersonal care processes [17,18]. Clinical care refers to the application of medical and other sciences and technology to achieve the best health result whereas interpersonal care describes the social and psychological interactions between health care professional and users of the health care system [17,18]. Outcomes, including patient satisfaction, are a consequence rather than a component of care and may be directly or indirectly influenced by the structure and processes of care [17,18].

### Sample and recruitment

Study participants were recruited from five urban centres across Canada: Vancouver, Calgary, Winnipeg, Hamilton, and Halifax. Each of these centres offers a range of prenatal care services to diverse populations. Purposeful maximum variation sampling was used to select informants that would provide a broad range of perspectives, thereby creating in-depth understanding of important dimensions of quality prenatal care [19,20]. Women were eligible to participate in the study if they were in the late third trimester of pregnancy (≥ 32 weeks), ≥ 16 years of age, and able to read and write English. We aimed for variation in age, parity, medical risk status, socioeconomic status, ethnicity, and urban vs. rural residence. Women were recruited from a variety of settings offering prenatal services (e.g., maternity clinics, hospital prebirth registration clinics, public health programs). Staff at each setting assisted in identifying potential study participants; women deemed eligible were given a study information letter and, if interested, gave signed permission to have their names forwarded to the site research assistant. The research assistant subsequently contacted women to further explain the study, answer questions, and arrange data collection with consenting individuals.

Prenatal care providers, including obstetricians, family physicians, midwives and nurses, were eligible to participate if they had practiced in obstetrics/maternity care for a minimum of 2 years. We tried to ensure diversity in characteristics such as profession, length of time in practice, type of practice (solo vs. group), and place of practice (urban vs. rural setting). The research coordinator sent a letter of invitation to care providers identified by study team members and followed-up with a telephone call to determine their interest in participating. Snowball sampling was then used as these individuals were asked to suggest other potential study participants.

### Data collection

A **s**emi-structured interview was conducted by a trained research assistant with each study participant at a location of their choice. Signed informed consent was obtained prior to the start of data collection. An interview guide informed by Donabedian's [17] model was used. The guide included an opening question, "What does quality prenatal care mean to you?" Then a number of questions were posed asking about structural aspects, clinical care processes, and interpersonal care processes perceived to contribute to quality care. Probes for each question were identified to promote consistency in data collection across study sites and participants. Women took part in a face-to-face interview late in the third trimester of pregnancy and a second interview was conducted by telephone approximately 4 weeks after they had given birth. This follow-up interview was an opportunity to obtain additional perspectives about quality prenatal care as women were in a position to reflect and comment on their care experience having had their baby. It also provided an opportunity to validate the themes emerging from the initial interviews. Care providers participated in a single face-to-face interview. All interviews were digitally recorded and transcribed verbatim. A brief sociodemographic questionnaire was administered at the end of the interview to collect background information on study participants. Women were given a $20 gift card in appreciation for their time and contribution to the study.

### Data analysis

The qualitative data were managed and analyzed using NVivo 7. We began the analysis using an inductive approach. The transcripts initially were read in full, with analysis then proceeding using open coding techniques whereby each meaningful segment of text was assigned a conceptual code [21,22]. A research assistant had responsibility for the coding and met with the principal and co-principal investigators after coding the first few interviews to develop a preliminary coding scheme, which subsequently was applied to the remaining interviews. Through comparative analysis, the same codes were assigned to data with common characteristics [22]. As the open codes became saturated, the analysis evolved to pattern coding whereby specific dimensions of quality prenatal care were identified [21]. Finally, a deductive approach was used to assign the emergent themes to broader categories that reflected Donebedian's model [17] and its further elaboration by Campbell, Roland, and Buetow [18]. The research assistant and principal investigator met periodically throughout the coding process to discuss and revise the coding scheme and the synthesis of codes at a higher level. The quantitative background data were entered into and analyzed using SPSS 17. Descriptive statistics were used to summarize the data collected from all women and prenatal care providers.

A number of strategies were used to ensure rigor of the qualitative analysis. In addition to the involvement of the principal investigator in coding, the emergent coding scheme was discussed with the co-principal investigator. Data were reorganized as the coding scheme progressed and all themes were firmly grounded in the data [23,24]. Memos were kept about coding decisions along with copies of evolving coding schemes [25]. Having women validate the emergent themes further enhanced the trustworthiness of interpretation of the interview data.

## Results

We recruited eight pregnant women and eight prenatal care providers from each of the five study sites, for a total of 80 participants. The sociodemographic characteristics of women who participated in the study are shown in Table 1. Five women (12.5%) reported they had experienced a pregnancy complication and eight (20%) had a physical or mental chronic health problem. Twenty-four women (60.0%) had seen an obstetrician for prenatal care, 21 (52.5%) had seen a family physician, and seven (17.5%) had seen a midwife; 30.0% (n = 12) reported receiving care from more than one type of provider. Twenty-seven of the women (67.5%) who took part in a prenatal interview participated in a follow-up interview at 4 weeks postpartum. Of the prenatal care providers interviewed, 14 (35.0%) were midwives, 12 (30.0%) were family physicians, eight (20%) were obstetricians, and six (15.0%) were nurses/nurse practitioners. The majority (78.0%) of care providers was practicing full-time and most were female (80.0%). The mean number of years in practice was 16.1 years (SD 11.8).

**Table 1:** Characteristics of women study participants (n = 40)

Characteristic	
Age in years (mean ± SD)	30.4 (6.5)

	**n (%)**

Marital status	
Married	25 (62.5)
Common-law/living with a partner	11 (27.5)
Separated/widowed/divorced	-
Single (never married)	4 (10.0)

Annual household income	
No income	1 (2.5)
< $10,000	-
$10,000-19,999	4 (10.0)
$20,000-39,999	3 (7.5)
$40,000-59,999	10 (25.0)
$60,000-79,999	3 (7.5)
≥ $80,000	19 (47.5)

Country of birth	
Canada	32 (80.0)
Other	8 (20.0)

Language spoken at home	
English	36 (90.0)
Other	4 (10.0)

Highest level of education	
Some high school	2 (5.0)
Completed high school	8 (20.0)
Some community college/technical school	2 (5.0)
Completed college/technical school	-
Some university	5 (12.5)
University degree	23 (57.5)

Table 2 summarizes the themes that emerged from the data analysis and their relationship to the predetermined categories that are reflective of Donabedian's model [17]. An additional theme, meaningful relationship, was woven throughout the interviews and cut across the three categories. While each group of informants emphasized different elements of quality prenatal care, the groups were similar in their overall views of what constitutes quality care. Therefore the data from both pregnant women and care providers were combined for the analysis. In presenting the findings, we use quotes for illustrative purposes. The source of each quote is indentified, with "*W*" and "*PCP*" being used along with study site and participant numbers for women and prenatal care providers, respectively.

**Table 2:** Quality of care categories and themes

Categories^1^	Themes
Structure of care	Access
	Physical setting
	Staff and care provider characteristics

Clinical care processes	Health promotion and illness prevention
	Screening and assessment
	Sharing of information
	Continuity of care
	Non-medicalization of pregnancy
	Women-centredness

Interpersonal care processes	Respectful attitude
	Emotional support
	Approachable interaction style
	Taking time

^1 ^Based on Donabedian's model of quality care

### Structure of care

Structure of care reflects the attributes of the care setting that contribute to the quality of care. The themes include access, physical setting, and staff and care provider characteristics.

#### Access

Access is defined as "the potential ability of women to enter prenatal care services and maintain care for herself and fetus during the perinatal period" (p. 220) [26]. According to study participants access includes being able to begin prenatal care as early as possible with a health care provider of the woman's choice. Access also encompasses having care available in locations that are convenient to women's homes or places of work, close to bus routes, and with adequate free or inexpensive parking. As women reported, *"It's actually quite convenient 'cause I can walk there [from work] on the nice days. ... It's close to my husband's work as well"(W03-04) *and, in contrast, *"We're spending over $60 a month just for parking [for prenatal care]. It would really make a difference if there were some options in that respect." (W04-04)*

Ease of scheduling appointments and office or clinic hours that are flexible enough to accommodate women's personal lives were identified as dimensions of quality care. A family physician spoke about this flexibility as follows:

Families come and mothers-to-be come in all sorts of abilities to organize their lives and get to appointments and follow through on stuff. So I think to have some flexibility around sort of how you, how you offer them care is, is really important and I must say we're probably way more flexible with our prenatal patients and our new moms. ... It doesn't matter if they show up late, it doesn't matter if they miss three appointments in a row. You know they're not penalized for that because you're really trying to sort of hold on to that relationship and, and build on it as opposed to sending them the little letter about one more no show you're out of here. (PCP04-08)

Women, in particular, commented on the value of having prenatal care providers available to them outside of scheduled appointment times. They identified that they often had concerns or questions they felt either were too serious to wait until their next prenatal care visit or did not warrant a visit. Having telephone access to the care provider or staff and having phone messages returned promptly were identified as important in reducing anxiety and feeling cared for. One woman commented:

I think being able to call and get somebody to call you back in about 10 or 15 minutes has been really great. I think that - I don't know that I wouldn't have had as healthy a pregnancy - but I think I would've felt a little bit more stressed out about certain things. (W01-06)

Women extended the notion of access to include access to educational resources. Ready availability of a variety of pregnancy-related educational materials such as books, pamphlets, and videos was another important component of quality care as reflected in this statement:

The rooms are good because you looked around and you saw everything that your future was going to be and you had access to pamphlets, you had any questions you'd have anything. Everything was there for you. Everything - you were completely surrounded. ... It's there and that's the greatest I think. Everything is available to you. (W04-01)

#### Physical setting

Women described several physical features of the setting in which they received prenatal care that contribute to its quality, including cleanliness, aesthetics, and privacy. They noted that the latter was particularly vital when providing urine samples and communicating with their prenatal care providers. One woman remarked on the importance of discussions that cannot be overheard by others as follows:

Privacy ... I wouldn't want to be sitting in the waiting room listening to a doctor speak with a patient. So it's reassuring to me to know that if I can't hear him talking to someone then nobody can hear him talking to me. (W05-01)

Prenatal care providers similarly recognized the importance of privacy. One health care professional compared differences in privacy in two different settings in which she had worked:

Compared to our old clinic, which was very small, very crowded, there is a lot of privacy in this clinic, which I think is important. You know, once you come into the back room, people aren't really seeing you, whereas in our last clinic there was a lot of movement in and out, and you could see people getting their blood pressure done and all that. And you know, that's okay for some people, but it's, you know, it's not okay for everyone. ... Not everyone wants to have their belly measured in front of a hundred people. (PCP02-05)

Having the right type of seating was important as it was noted that pregnant women often have back problems and thus find it difficult to find comfortable seating. The aesthetics of the physical space also were identified as contributing to quality care. As one woman stated:

I like how this whole building obviously, it's different colours. It makes it a good atmosphere. It's very clean. ... You get an overall happy feeling when you're there. You're not sitting in a dark office, gloomy feeling. It's bright and everything's bright, makes you feel happy, I find, so I like the different colours and stuff like that. (W05-05)

Women desired a "*welcoming*" environment and often described family physicians' or obstetricians' offices as "*medical*" and "*clinical*" whereas those of midwives were described in more favourable terms, as captured in this quote:

She's got a very nice chaise lounge rather than an examining table, and then she has a little desk. ... So it's pretty comfy. And then there's a separate room for pelvic exams. Yeah, the whole place is kind of upholstered and furnished. It does not feel clinical. There is a minimum of rubber gloves and paper, and cold metal stirrups and whatnot. (W01-07)

#### Staff and care provider characteristics

Women discussed how the characteristics of staff who work in prenatal care settings contribute to its quality, including the temperament and personality of office staff. Staff who were pleasant, greeted patients by name, and were efficient had a positive impact on how women viewed their care. As one participant commented:

I would say that it's [the environment] a positive one because she [the receptionist] greets me with a smile, and again, non-judgmental, even if she's really, really busy, she doesn't act like she's flustered or stressed out. I think that's really important because people's energies can impact another person. And it could ruin your day. So if she had a bad day, it could ruin my day too because she just yelled at me or something like that. (W01-08)

For some women, knowing that their prenatal care provider had clinical experience and was not new to providing prenatal care engendered a sense of confidence in their abilities, as conveyed in the following remark:

They definitely exude a kind of a relaxation and a confidence about what they're doing. All the tests they do, they make them seem so easy. I think they've been doing it so long, they know exactly where the baby is. They know exactly how to get a heart rate. ... You know that they know what they're doing, so it's relaxing to just go in and let them do their thing and know that everything's fine. (W02-05)

Many women suggested that a prenatal care provider's clinical expertise is enhanced by having personal knowledge of pregnancy and childbirth. One participant explained:

I feel like I can relate with them, and they have some kind of vast experience. ... You always feel more comfortable with the person with more experience. And I feel that they [prenatal care providers] have a lot of experience, especially because they have children of their own. (W02-08)

Health professionals also spoke more about the contribution of clinical expertise to quality prenatal care. One family physician noted this as a special consideration when transferring care:

I have one obstetrician/gynaecologist in particular that I refer to who I worked with during my training, who I have a relationship of trust with. So I can say with confidence to the people who I'm sending on, "I trust this woman, she's great, she's clinically sound, she will take really good care of you." (PCP03-04)

### Clinical care processes

Clinical care processes denote the application of clinical medicine and knowledge-based care [18]. We expanded this definition to include a patient-oriented approach to care with active involvement of women in their own prenatal care. The themes for clinical care processes are: health promotion and illness prevention; screening and assessment; information sharing; continuity of care; non-medicalization of pregnancy; and women-centredness.

#### Health promotion and illness prevention

Women and prenatal care providers identified the importance of health promotion advice to encourage a healthy lifestyle. A family physician talked about taking time to address smoking in pregnancy as follows:

If they're smoking I spend quite a bit of time addressing the concerns about smoking because I really try and emphasize the positives of them not smoking when they're pregnant and then hopefully then towards the end push the need to be a non-smoker afterwards. (PCP05-04)

Counseling about nutrition and appropriate weight gain was identified as an essential component of quality prenatal care. An obstetrician described her approach to this as follows:

I think I do far more nutritional counseling than most people do because I do watch their weight gain and I do try to get them to do 3-day food diaries and to try to help them to bring the weight under control or the weight gain under control if it's getting a bit out of control. So I have Canada's Food Guide that I tell them to take and I consult nutritionists if necessary. (PCP05-05)

Women commented favourably on the impact of receiving health promotion advice. One study participant remarked on the insight she gained from her prenatal care provider asking her to record her food intake:

Well, definitely you have to eat properly. ... I know I didn't know the proper amounts of different things you need during the day. ... They had one that was a check list. And you could check off every time you ate this and how much of this you ate, which I found really helpful 'cause you don't realize what you're not getting until you have this thing in front of you. So it definitely helped me out. (W05-05)

For other participants, their care provider's advice encouraged them to maintain a healthy lifestyle. As another woman explained:

Well, it [advice] encourages me to keep doing what I'm doing. Like move around instead of getting lazy or giving up or anything. ... Like no matter how lazy I am, to still get off the couch and get some exercise. Also 'cause that's what's keeping them healthy is the exercise and the nutrition and everything. So just the encouraging me to do extra things and just keep going with my regular life is the major thing. (W04-08)

Health care providers commented more frequently than women on the need to pay attention to known risk factors during pregnancy as an illness prevention strategy. The factors most commonly discussed were preexisting health problems, health-risk behaviours, and social risks to health. One obstetrician described his approach to risk assessment as follows:

I ask her if she has any known medical problems and then, I must say, I coach a little bit because many people don't know what a medical problem is and I usually name diabetes, heart disease, kidney disease. If she's ever had tuberculosis, epilepsy, rheumatic fever, hepatitis.... I ask her if she smokes, if she drinks, if she's ever had street drugs. (PCP05- 03)

Women similarly noted the importance of attending to preexisting health conditions and as one participant remarked, "*That's an important part of the prenatal care". (W04-06)*

Some care providers spoke about assessing social risks to health and linking women to appropriate resources in their communities. As a family physician stated:

They [women] might bring up that they've got some anxiety or whatever. And I just like to make sure I delve into that because you uncover things like physical violence and financial issues. ... I think if you take the time to ask those, like a few questions about how things are going in life, and get a sense of where people live and how they're living, you do pick up on a lot of those extra things. ... And we can give them places where they can go get support for that ... making sure you get social workers involved appropriately, especially if you're a woman at risk of physical violence. (PCP02-05)

#### Screening and assessment

Both women and health care providers discussed the value of screening and assessment as part of quality prenatal care. Women talked primarily about tests and measurements that provided reassurance the pregnancy and fetal development were progressing normally. As one woman commented, "*Every time they check me really good so I'm satisfied that my baby is doing good so and even I'm doing good. They check my blood pressure, my diabetes, my protein and everything." (W02-03)*

Prenatal care providers highlighted the importance of following guidelines for screening in pregnancy to ensure better outcomes for mothers and babies. An obstetrician noted:

The things I really think improve outcome are screening tests for HIV, screening for Rh disease. Rubella immunization, we still do it. And syphilis, although it's low probability of picking something up, it makes a big difference when you do. The screening ultrasound, whether or not that's making a big difference in the outcome, I know, is a little debatable. We try and follow up on those guidelines.... So we do a fairly structured, follow all those guidelines for screening for group B strep. I think those things are really important in changing outcomes. (PCP02-01)

Health care providers also discussed the importance of screening and assessment related to psychosocial health as reflected in the following comment made by an obstetrician:

... assessment of how the woman's doing with relation to her pregnancy and in relation to her life and are there any arising medical conditions that might impact on her pregnancy? Or social conditions. ... It may be that if she's under undue stress that there may, that there may be ramifications in terms of how she's coping with the pregnancy or how she's going to cope with it once the baby's delivered. Her risk of postpartum depression is likely to be increased by increased life stressors during the pregnancy and in the postpartum period. So I think it's important for me to know about that. ... I can be supportive and at least I know that that woman's at risk so that if she starts to go downhill I can identify it and get perinatal psych involved for example. (PCP05-05)

Screening and assessment were identified as being essential as a first step in ensuring women receive appropriate care not only for mental health problems but also for physical health concerns that develop during pregnancy. One midwife, for instance, discussed hypertension and the importance of medication in its treatment:

Hypertension might actually be managed to improve outcomes. ... Because the way in which we manage hypertension now is quite different than the way in which it was approached say 30 years ago and so we don't see the same sort of things. I mean we don't see eclamptic women. I can't remember when the last time was I saw somebody seizing because of their high blood pressure, right. We medicate people differently, we treat them differently, and I think their outcomes are likely to be better. (PCP04-01)

#### Sharing of information

The sharing of information by health care providers was identified as a key aspect of quality prenatal care, particularly by women. When asked what aspects of prenatal care were important to her, one woman replied:

I believe it's the way they involve you, and the way they tell you everything that's going on. So there's no secrets, there's no mysteries, there's no secret codes or anything like that that you don't understand. ... It makes you feel like you are totally in the loop and you know just as much as the doctors know. ... And it makes you more confident, and like more prepared, and just feels good to know everything that's going on. (W02-06)

As suggested by the remark about "*no secret codes*", it is important that information be conveyed in a way that women can understand. Another woman commented:

Like I understand what she's talking about when she talks to me. And I think she tries to make it that way. I don't leave, ever leave and kind of go, "What did she say? What is she talking about?" You know? So that's been good. (W01-03)

One woman expressed how much she appreciated being given information about every aspect of her prenatal care visit:

The nurse will let you know whether your urine was good or if there was a problem. And then she'll check your blood pressure ... she'll give you the number, and then she'll explain what it is, whether it's good or not. And then she weighs you and then she tells you first what it is, and then how much you've gained or lost or anything like that. She is very, very specific and detailed about letting you know how you're doing that way. ... It makes me feel involved. It makes me feel confident. It makes me feel like they actually care and they pay attention to what's going on. (W02-06)

Women expressed the value of health care providers sharing information in an open and honest manner, even when delivering potentially distressing news. One woman described her experience as follows:

They also have a way of breaking it [information] down so that it makes sense to people who don't have medical degrees and help you to understand the ramifications. For example ... the last appointment I had with my endocrinologist, which was a week and a day ago. And we were talking specifically about the ramifications of a larger baby. And he didn't beat around the bush. He just told me upright what they were. First trimester, second and third trimester. So, that was kind of nice as well ... because then we know what we're dealing with, with this larger size baby and because then we're more prepared. (W04-04)

Health care providers also commented on honesty as an element of quality care. As one obstetrician stated, "*I try to build confidence in our relationship. I am completely straight forward. Nothing is dressed up." (PCP05-05)*

#### Continuity of care

Many women explained that receiving care from the same health care provider throughout the pregnancy was a feature of quality care. Some noted that this allowed the health care provider to be familiar with and effectively monitor their pregnancies. One woman who experienced several care providers explained:

I see almost six doctors. ... Every week I saw different doctors. ... When you go and see different doctors maybe they didn't know our progress. ... When you have some problem it's necessary to clarify all this for all new doctors. ... It's better to see one specific doctor all the time because they see everything, our progress and otherwise if I saw every doctor, every different doctor - it's not good I think. You get to know them better. And if you have issues from one, you know, week to the next, you can say, "Well, how are your feet today?"or "How are your hands today?" and "Do you have any other symptoms?" ... It's all about just continuity and rapport, relationship. (W02-01)

Other women elaborated on how having a consistent care provider throughout the pregnancy contributed to the development of a positive relationship. When asked if she felt continuity of care was an important feature of quality prenatal care, one woman responded:

Yes, I think it would be because you, you build the relationship. They know.... They're from there from the beginning. Right when it's like a little kidney bean instead of two little babies, right? So, you're gradually growing the baby together rather than half way through you're getting to know the doctors again. (W04-08)

Some women commented specifically on the importance of receiving prenatal care from a health care provider with whom they had developed a relationship, as exemplified in the following remark:

I think it's more of a personal thing with the doctor. Like my regular doctor, I've had since I was eight. So like going in to see her - it's sort of like going in to see someone you've known forever. It's, "Hey, how's it going?" You know, you have your little chit-chat first and then you get into what's going on. Whereas when you're go see someone new, it's kind of - I don't know if you're going to be embarrassed about things, or you're kind of shy about talking about certain things. (W01-03)

When women had different prenatal care providers during their pregnancies, they identified a smooth transition between care providers and timely, efficient sharing of information as important factors in continuity of their care. In reflecting on her transfer of care from a family physician to a midwife, one study woman observed:

Transition wise it would be helpful if there was a bit more of a flow. Like something where you know the GP understands ... she just kind of said you've got to go find care and I was like okay and then I sort of felt like, well, am I supposed to put them in touch or do they talk? Or is there like my medical history? Is that relevant to the pregnancy? ... At one point of course my midwife had to request some of my relevant medical files. Like had I had rubella shots etcetera. Things like that. (W01-01)

#### Non-medicalization of pregnancy

A recurring theme in the study was the importance of not treating pregnancy as a disease or medical condition, but rather as a normal process. When asked about her impressions of prenatal care, one woman responded:

I just thought there'd be more, it would be more medicalized, which I'm glad it's not, actually. You know?'Cause you want to feel like a normal person, too, even though you're pregnant. You don't want to feel like a patient. (W02-04)

Several women expressed a preference for receiving care from a midwife because it felt less medically-oriented than care provided by physicians. One woman contrasted her experiences as follows:

From the first appointment that we had with our midwife I always said I just loved that it didn't feel as medical and as clinical. Like it didn't feel like I was sick when I went to see her. And when I went to see my family doctor, although I didn't actually feel like I was sick, I just felt like I was going in there for like a medical condition. Whereas with my midwife it was just sort of this experience we were all kind of having. And it sounds a little cheesy and hokey but that's kind of how I felt. (W03-04)

Some health care providers, particularly midwives, also identified the importance of not treating pregnancy as a medical condition. A midwife emphatically stated:

Pregnancy - pregnancy is not a disease [banging on table]. Pregnancy is a normal physiological state. Women become pregnant and you shouldn't make a pathology out of it. It's not an indication to stop work. Pregnancy is a condition of moderation. You want to exercise, you exercise. If you are a skier, ski but maybe stay off the black double diamonds and do the nice blues and do something that you're comfortable with. Don't get over tired and I think that that's fine. But a lot of people try to make pregnancy a disease and it really isn't. (PCP05-03)

One physician similarly commented, "*The traditional visit is the doctor and the patient and their partner.... It kind of medicalizes their pregnancy, which for the most part isn't really necessary." (PCP01-06)*

#### Women-centredness

Women-centredness emerged as a salient attribute of quality prenatal care from the perspective of both women and health care professionals. Key principles of women-centred care are that it situates care within women's life contexts, acknowledges the social determinants of health, and positions women as active partners in their care rather than as passive recipients [27].

It was evident that women valued a high level of personalization in their prenatal care. They wanted their prenatal care providers to pay attention not only to their pregnancies but also to the psychosocial aspects of their lives. Health professionals similarly noted the importance of "*practicing comprehensively*". As one midwife stated:

I try to focus not only on the medical stuff, but the social and to some extent cultural. I try to focus and not just think only about the pregnancy, which I think is a little bit different about the way we practice compared to someone who's practicing high risk. ... We like to think of ourselves as taking care of the entire patient and not just focusing on the pregnancy. (PCP02-05)

Some prenatal care providers spoke specifically about the need to attend to priorities created by women's life circumstances as reflected in this family physician's comment:

I obviously am supportive of health promotion and education but I'm cautious that it doesn't undermine the sense of the woman being an adult and having other priorities in her life ... one size doesn't fit all. ... We tailor the education and health promotion to suit the woman's particular chapter in the life she's in right now. For example, a woman is leading a devastated life. Isn't quite sure where she's going to sleep at night. Now is not a good time to talk about quitting smoking. Her priority is safety and things like that. (PCP03-03)

Consideration of women's life circumstances extended to allowing women to choose to include significant others, such as partners or other family members, in their prenatal care. This was conveyed in the following statement:

Both of the doctors that I've seen have been really great about ... encouraging the father to come to, or whoever, a parent or a friend to come. And actually, what was nice, the first time or the second time that I had an appointment with the doctor who will deliver me, she knew my fiancé was in the waiting room. And she said, "Oh, bring him in here because, he's a part of this and I want to meet him. He should be a part of this experience as well." So that was nice. Having doctors who ... treated it as more of a family experience. (W01-05)

The involvement of women as active partners in their care was recognized as an essential feature of quality prenatal care by most study participants. A midwife described how involving women can foster positive health promoting behaviours and outcomes:

And the medical course of events becomes not something that happens to a woman, but something that she is part of making happen and having happen. ... I think if a person does feel very involved in their own prenatal care ... that does encourage their motivation to be as healthy as possible. And that could certainly make for more positive outcomes. (PCP01-05)

The main aspects of women as active partners included giving them responsibility for routine aspects of care. Another midwife commented on how this can engender a sense of control:

We have a little set up where they check their urine. The cups are there right at the bathroom and the scale is right there. When you do that you're saying to women, "We totally trust you."... If you're not colour blind you can read a urine strip, it's a no brainer and when they take their own blood pressure. So they take charge for their body and they hand over to us to measure the baby and check that well being. And I really like that model. I think it sends a message. (PCP01-03)

Women concurred that such involvement gave them a sense of control, as reflected in this statement:

You weigh yourself, and you check your own urine as far as just protein and sugar. ... And just let them know the colour of the strip, and let them know what you weigh. So you are kind of monitoring yourself a little bit, which is nicer because you feel in control. (W02-07)

Women's active involvement in their own care also included meaningful participation in decision making. In one woman's words:

Straight off the bat, seeing her just to confirm my pregnancy, she was very reassuring about taking charge of the medication that I was on, should I stay on it, should I go off it, and sort of consulting with me and what I felt comfortable with as well as her medical opinion. And that was really, really helpful, that I was involved in the decision making but I knew that she knew what she was talking about. (W04-06)

Health care providers agreed that giving women information and allowing them to make informed decisions was important. As a midwife remarked:

The goal of that [informed consent], the women, the families, feel like they're making really thorough decisions for themselves. That puts them in charge of their own care. ... We try hard not to direct care, except in times when we feel we have more information than they can have, just because we are care providers. So of course there are times when we will say, "You know what? I think you really need to have an ultrasound for growth", and that's going to really make me more comfortable. But for the really routine stuff, it's important that women can feel they direct that themselves, and also feel ownership over it. (PCP01-04)

Another midwife explained how involving women in decision making throughout their care engenders trust in a care provider when s/he needs to be more directive:

Trust needs to be built, because here I am bringing somebody in saying, "You have some responsibility in your care. I'll help you make decisions and I'll give you the education you need. And of course I see myself as having played a huge role because I'm the care provider, but you have a responsibility to go home and read and educate yourself about whatever it is I'm talking about. And then we'll make the decision together. But you're the one who's really making it in the end. You're running the show." And so in order for them to actually trust themselves to do that, they have to trust me first, that I'm going to be able to say to them, "Well, I hear you that you want to have a home birth with your triplets that are all breech but actually that's probably, in my opinion, not the best idea." (PCP01-08)

### Interpersonal care processes

Interpersonal care processes reflect the psychosocial aspects of interactions between prenatal care providers and the women to whom they provide care. The themes are respectful attitude, emotional support, approachable interaction style, and taking time.

#### Respectful attitude

According to study participants quality prenatal care involves a respectful regard for women as care recipients. Women expressed a desire for care providers who are non-judgmental and who therefore are easy to talk to. In one woman's words:

She's very easy to talk to. Just like talking to a friend more like, she doesn't seem to judge you or anything. It's easy for me to open up especially when I had troubles talking to doctors. Well I thought I had troubles talking to doctors. (W03-01)

Several women specifically noted the importance of their prenatal care providers not minimizing their concerns or making them feel foolish when asking questions. One woman described her experience as follows:

I guess one of my worries was that, you know, sometimes because this woman [care provider] sees so many pregnant people that she might kind of downplay my concerns. And I think that would make me feel uncomfortable and reluctant to share some of my feelings. And that hasn't been the case, and I definitely felt like I can call or bring up these concerns and to not feel stupid about it. So that's been, I think, good at relieving my stress about the whole experience. (W01-05)

When asked what characteristics were important in a prenatal care provider to enhance quality of care, health care professionals also discussed the importance of being non-judgmental. As one obstetrician responded, *"The big one's non-judgmental. So you have to be able to, you know, care for them whatever they bring in. And you get really good at not letting your jaw drop when they tell you things."(PCP03-05)*

Many participants noted the importance of women feeling respected and valued at all times. A very young mother shared, "*Oh they treated me good. Like they were really respectful and they, I don't know, they were never rude or treated me bad just because of my age. I just like to feel like everybody else, like to feel respected." (W03-06) *One midwife shared some thoughts about respect saying:

I don't really know that it [quality care] has much to do with prenatal care but just care in general you know? It's about respect for people. ... It's about respecting their intelligence, their understanding of the world. ... It's about respecting their ability to make good decisions about themselves and for themselves. (PCP04-01)

Included in this theme is acknowledgement of and respect for cultural differences. Some health care providers spoke of the importance of offering services in a culturally sensitive manner. A family physician stated:

A significant number of our patients are from a different culture than the care team - African or Asian or First Nations. I think we are reasonably successful to provide care across that cultural divide. It has mostly been my experience to work to do that, and I think it can be successful. But obviously, it takes some sensitivity. (PCP03-06)

#### Emotional support

What emerged as one of the most essential features of quality prenatal care was the provision of emotional support, which is conveyed through behaviours such as listening, expression of caring and concern, acknowledgement of feelings, and reflective understanding [28]. Nearly every study participant, both women and health care providers, talked about its importance. One woman described an example of emotional support and the effect it had on her as follows:

The one where my blood sugar was higher was actually really good in an odd way because I had gone in and I had my crazy morning at work already and when I got there I tried telling them and I just started crying. Apparently it's the hormones so she [obstetrician] just stood up and gave me a big hug and I just felt like laughing. It was kind of nice. (W01-01)

Women also spoke of needing to feel that they and their pregnancies were important to their care providers. For one study participant, this was conveyed through a family physician's understanding of the significance to her of hearing the baby's heart beat:

I met a doctor there and he was just so warm and fantastic, and I was almost 8 weeks pregnant and hadn't heard the heart beat or anything yet. And he said, "Hey, let's listen to the heart beat. Go grab your husband from the waiting room." And it was just incredible. Because I was like - "Oh my god! We're gonna hear the heart beat!" And it's your first pregnancy, and it was just like he went above and beyond. ... He was just really warm and exactly what you want in a pre-natal care provider. (W02-07)

Women wanted "*to just feel cared for" *and have reassurance from their prenatal care providers throughout their pregnancies that their babies' development and pregnancies were progressing normally. One woman expressed:

I think for me the most important aspects would be knowing that I'm okay. So knowing that my blood pressure's okay. And knowing that the baby's heartbeat is - I can hear it, and it's same as always. ... And knowing that, say for instance, the size of my uterus is the average size of everybody else's uterus, right, so at this time of pregnancy. So I would just say kind of being reassured that all my vitals, the baby's vitals are all fine. (W02-04)

An additional feature of emotional support was acknowledgement of women's feelings and reassurance that they were normal. One woman who did not receive the emotional support anticipated from her midwife reflected on her disappointment as follows:

I had thought that taking this route would give me a bit - like an element of that emotional support - just that little bit of emotional reassurance. ... I get the clinical reassurance regularly but the emotional reassurance that no, you're not crazy and you know the crying or whatever's happening is totally normal. You'll be fine. That element has been a little bit lacking but that might have been a false expectation on my part. (W01-01)

Prenatal care providers similarly recognized that listening and providing reassurance were key aspects of their roles. One family physician commented:

I just try to alleviate stress and concern. People worry lots about everything and things that are out of their control, so I try to just be laid back and make sure they know that things are okay. I'll worry about things that need to be, and just lay back and enjoy yourselves - look forward to the baby. I find prenatal care very easy and very relaxing. Most patients, they just want to know that they're doing well and that the baby is healthy. So you just have to reassure them. (PCP03-08)

#### Approachable interaction style

Interaction style refers to behaviours that characterize the manner in which health care providers carry out their responsibilities [29]. Women expressed a preference for prenatal care providers who are positive and engaging as suggested by this comment: *"This is my second go-around so we've had her for both pregnancies and she's very positive - always makes you feel very comfortable and even uplifted during that visits, which is nice." (W05-04)*. The use of humour was identified as an effective strategy to engage women. One participant stated:

I think it [humour] just puts people at ease. You know if everything is very serious and to the point all the time you get a little more tense. If you can laugh about things then you know it just puts you at ease. You're a little more relaxed. (W04-07)

Several women commented that it is important for their prenatal care providers to be calm and relaxed as this reportedly helps to reassure women and to engender confidence in the care provider. Additionally, these characteristics made care providers "*approachable*" in that women felt comfortable asking questions. As another woman expressed:

I have an excellent relationship. I really like my obstetrician. ... She's fairly laid back and she doesn't make me feel uncomfortable or nervous about asking all the questions that I have and I feel very confident in her experience. (W05-07)

Health care providers also acknowledged the need for a calm and relaxed demeanor. One obstetrician remarked:

I find that when something bad is going on, it doesn't help things for you to be excited, and anxious, and worried, and what not - especially to verbalize that or show it to the patient, because that just makes it worse. So you have to be a little bit more that calming voice, supportive, reliable and efficient - do what you have to do. But you know raising all the fire alarms is just going to make it worse. (PCP03-08)

#### Taking time

Women placed considerable value on the amount of time their health care providers spent with them during prenatal visits. They identified the importance of care providers taking time to address all their questions and concerns. One woman recounted her experience as follows:

I really like that they take the time for me to just go through my list of questions. I don't feel like I'm wasting their time or that it's boring. I can just sit there and go okay, "What about this? What about that?" And they don't mind that - that's fine. So that I would say is the best part of it - is that I have the time to ask my questions. (W01-06)

Women clearly did not want to feel rushed during their appointments. When prenatal care providers appeared to be in a hurry, some women reported they did not have adequate opportunity to formulate questions, as captured in this remark:

My expectation would be that of course, the doctor would just ask, well they do ask, "Do you have any questions."And usually the answer is, "I don't know yet." And as soon as you don't answer within two seconds, okay, see you next week. Maybe it would be nice if the doctor would like, at least wait ten seconds to give me a chance to formulate my question. 'Cause sometimes what I do, in the past, was have a list of questions and then go there. But then, um, they're not happy because there's like ten questions, which is using up all their time. I feel a little bit guilty, having too many questions. (W01-08)

Several women expressed a desire for longer appointments, especially at the beginning of their pregnancies when they had more questions or when important decisions had to be made. One participant commented:

That first prenatal appointment, there is so much to go over. ... Like I said, the genetic screening, the various blood tests ... all the prenatal testing that is done now, there is just so much to give. And a lot of prevention and sort of advice on what to avoid and what's okay, and what's not okay. I think a lot of that gets missed by the doctor because they don't have time. (W01-02)

A number of women identified a difference in the amount of time spent with midwives compared to that spent with physicians. Many of the women stated a preference for midwives because of the length of each appointment. One woman noted:

You can be comfortable to ask all the questions you need to ask, and get the answers that you need to get, and now that they've [midwives] taken the time to really think about, or do the research to find out, and make sure that they're giving you the accurate answers. And I have found myself with the [midwifery] team that I'm with right now, I'm not having any problems with that. But earlier in my pregnancy with my family doctor, I did very much feel that they were kind of rushing through it all because they're just so rushed on time and appointments. (W02-05)

Some women had different experiences with their family physicians in that they did not feel rushed during their visits. As another woman remarked:

Sometimes when I've had some concerns, and when I thought about it later, I thought, "Oh my god." ... Months down the road I think, "Oh that was so ridiculous." But he never said to me, "Oh, don't give it another thought. You're crazy. Go home and relax." He never says that. He always listens to me. Um, doesn't rush me. (W04-02)

### Meaningful relationship

A recurrent theme throughout the interviews that cut across all three categories reflects what may be the very essence of quality prenatal care, a meaningful relationship between the care provider and the expectant mother. The relevance of the relationship to quality care is exemplified in this remark made by a woman who was experiencing her sixth pregnancy:

There have been things about each one [pregnancy] that have been different. And so to be able to comfortably discuss things with her [family physician] means everything. It means that the experience has been positive when I feel safe talking to her, and I feel like I can trust her. I feel like it's private, you know, our conversation. I feel like she's going to give me an honest answer about things, that she's going to be fair in her presentation about different options. And so having that kind of relationship has been really important to me in terms of feeling like I've had incredible quality care. (W05-08)

Other women similarly noted that a meaningful relationship is characterized by trust and as one woman commented, a relationship based on trust can reduce anxiety and ultimately contribute to positive outcomes:

I feel like I trust them, I feel comfortable and safe with them. I feel that they are both very well-trained and very experienced. I think that my anxiety will be lower because of the relationship that we have with them and that's got to have a positive outcome. (W01-07)

Having a meaningful relationship with a care provider also contributes to women's comfort in asking questions and becoming involved in directing their care. As one woman recounted:

I didn't know him [obstetrician] very well so you're walking in and it almost feels like a stranger because it's the reality, you're meeting for the first time. ... I didn't say very much. ... And then you start to build the relationship and that's where the difference is. And he was a great man, to be honest with you. He comes up to me with a smile all the time so, and he knows me by my first name. It does help you build that relationship. ... And seeing and having all that information [on warning signs] made me ask the questions that I needed to ask in order to have the proper information. (W04-01)

Health care providers recognized the centrality of a meaningful relationship with their prenatal clients to quality prenatal care. As one family physician remarked:

What are the aims of our prenatal care? I think also it's an unfolding process of a relationship. You know it takes you know the relationship that grows and develops between client and caregiver as well as just mother becoming a mother and a family becoming a family. Right? So and that, that's part of prenatal care and I think it's integral actually to prenatal care. (PCP01-01)

Health professionals, too, associated such a relationship with trust, comfort, and a reduction in anxiety for the women. Some care providers believed that the relationship they had with a woman played a role in her engagement in prenatal care. A midwife explained:

And so you build different relationships with different people 'cause some people are more forthcoming and that kind of thing, and other people are just doing their thing, right? But that relationship. I mean that's why women keep coming back. ... We've got people coming back who've had baby number four now. So, they keep coming back and that means you built the relationship. (PCP01-07)

Study participants, both women and care providers, acknowledged that a meaningful relationship makes it more likely that a woman will accept guidance and health-related advice. One family physician commented:

You have to find the right time to talk about that [smoking] too, and I think sometimes you have to develop a relationship first before you start saying you know this and I know this but is there any way we can help you reduce your smoking? Rather than walking in the room the first visit with your finger pointing, "I see you're smoking, don't you know that's bad for your baby and do you want to have a healthy baby or do you want to have a baby with cancer and asthma?"... Not a good bridge building. (PCP03-03)

A meaningful relationship between woman and her care provider therefore not only enhances the quality of prenatal care but also can influence the extent to which women adhere to professional recommendations.

## Discussion

The study findings provide information on the important elements of quality prenatal care as described by women and care providers, which reflect the structure of care and clinical and interpersonal care processes. There has been much attention in the literature to access to prenatal care, which is one dimension of structure of care. Our findings along with those of other researchers [30,31] suggest that convenience of care is a key consideration. This issue has been framed in the context of personal costs, including direct dollar costs (e.g., transportation costs) and costs of time (e.g., time away from work/school, travel time) [32]. We also determined that appointment flexibility, ready access to care providers by telephone, and access to educational resources are important in the provision of quality care.

Physical characteristics of the care setting deemed to be important to quality prenatal care identified by study participants have been reported in other studies. For instance, Proctor [30] found that cleanliness and homelike surroundings were quality indicators and in a study of group prenatal care privacy was identified as an issue by some women [33]. Staff characteristics also were noted to be elements of quality care. Women study participants commented on the importance of office staff who are pleasant and other research suggests that rude treatment by staff can negatively influence a woman's desire to return for appointments [34]. While both women and care providers remarked on the importance of a care provider's clinical knowledge, women additionally valued the knowledge and understanding practitioners gained through personal experiences with pregnancy and childbirth.

Clinical care processes and interpersonal care processes emerged as being most essential to quality care as discussions of these elements of care were far more prominent than discussions of structure of care in the interviews with both women and prenatal care providers. Care providers spoke of screening and assessment, a clinical care process, in terms of guideline adherence to ensure better perinatal outcomes. This perspective is congruent with discussions of the role of evidence-based care and guidelines in promoting quality prenatal care [12,13]. For women, these medical aspects of care provided reassurance about their health and their baby's health. Health promotion and illness prevention, including attention to risk factors, also emerged as components of quality care and are explicitly addressed in prenatal care guidelines [35-38].

Some of the other clinical care processes identified in the study have been identified previously in the research literature. For instance, the importance of sharing of information is captured in findings that women appreciate being offered information by clinicians [30,39], being kept well informed [40], and having their questions answered [41]. The importance of continuity of care provider also has been highlighted in a number of studies [30,39,42,43] and relates to women wanting clinicians to know them and to remember them from one visit to the next [40,42]. When women see more than one care provider, we found that sharing of information was valued as it facilitated a smooth transition. Some prenatal care guidelines specifically address continuity of care [35-37].

Sensitivity to women's life contexts or circumstances, an essential element of women-centred care, has been identified in other research [41,44] as has women's active involvement in decision making [30,45]. Professional guidelines often refer to a woman's right to informed choice. By way of example, the NICE guideline for antenatal care explicitly addresses informed decision making in stating that "pregnant women should be offered information based on the current available evidence together with support to enable them to make informed decisions about their care" (p. 12) [36]. Another key feature of women-centred care, personalized care, also has been noted in several studies [34,40,41]. Consideration of each woman's unique situation and needs provides opportunity for early intervention, particularly for risk factors associated with adverse life circumstances and socioeconomic conditions.

We additionally found that non-medicalization of a woman's pregnancy was a feature of quality care. In a study of group prenatal care, women appreciated that their pregnancy-related changes and fears were normalized [33]. Medicalization has transformed pregnancy and childbirth into an illness where there is an assumption of risk to fetal and maternal health that becomes the focus of prenatal care [46]. However, this orientation has been criticized because it creates dependency on medical care, undermines women's rights to autonomy, and minimizes the relevance of women's life contexts [46,47]. It therefore is not surprising that non-medicalization of pregnancy emerged as a component of quality care as many elements of quality identified in our study respond to these criticisms.

The interpersonal care processes revealed in our study as having a role in quality prenatal care included respectful attitude and emotional support. The importance of women being treated with respect has been noted in previous research [30,34,42]. Interestingly, only one of the prenatal care guidelines reviewed explicitly addresses this issue by stating that women should be treated with consideration and respect, and that the relationship between a woman and her care provider should be characterized by mutual respect [48]. Listening, which is an element of providing emotional support, has been highlighted as essential to quality care in other studies [14,30,44]. The value of clinicians who express caring is reinforced by a study that found ratings of antenatal care were higher if care providers were sensitive and understanding and women's concerns were taken seriously [40]. Caring also is conveyed through the expression of concern for and assessment of a woman's psychosocial well-being [49]. Importantly Sheppard, Zambrana, and O'Malley [44] identified that lack of caring and insensitive behaviour can deter willingness to follow advice and return visits.

The other two dimensions of interpersonal care processes, approachable interaction style and taking time, have received little attention in the literature. The importance of an informal interaction style was identified in a study in which women described their appreciation of a clinician's use of humour [41]. If women are put at ease and feel relaxed, they are more likely to engage with care providers, share information, and participate in making decisions about their care. Enough time with a care provider was identified as a marker of patient-centred care in another study of quality of prenatal care [14], and Davey, Brown, and Bruinsma [40] found that having adequate time with care provider increased overall care ratings of prenatal care.

Having a meaningful relationship with a prenatal care provider may be fundamental to quality care, and is inextricably linked with characteristics of the prenatal care provider and clinical and interpersonal care processes. In an integrated review of the literature on women's experiences of prenatal care, Novick [50] commented that the topic of relationships was discussed in the majority of studies, which further highlights its centrality to quality care. The notion of trust in the care provider was predominant in our participants' references to a meaningful relationship. Trust has been identified as a key indicator of quality in the patient-provider relationship, and having a trusting relationship with a care provider increases the likelihood that professional advice will be followed [44].

The strengths of this study are its exploration of quality of prenatal care across different settings, populations of women, and care providers. The large sample size of 40 women and 40 care providers ensured that we captured a broad range of perspectives and data saturation was achieved. Interviewer training included orientation to the study and its conceptual framework as well as practice interviews. Additionally, the transcripts of initial interviews were reviewed by the principal investigator and research coordinator, with feedback subsequently discussed with each research assistant. Strategies were put in place to ensure rigor of the analytic process and hence validity of the study findings. Study limitations relate mainly to the sample in that most of the women had been born in Canada, spoke English at home, and had a university degree; however, these characteristics do reflect the majority of women in Canada who have recently given birth [51]. Most of care providers were female, and there was an over-representation of midwives and an under-representation of obstetricians in the sample.

While intended to inform the development of items for the Quality of Prenatal Care Questionnaire, the study findings also provide direction for the planning and delivery of prenatal care. As noted, the findings reflect a number of the published prenatal care guidelines and, in particular, resonate with the Canadian Family-Centred Maternity and Newborn Care: National Guidelines [37]. These national guidelines recommend that: pregnancy be considered a state of health; women be valued and respected; the relationship between a woman and her care provider be consultative and interactive; and care providers facilitate informed decision making. Our study findings also are congruent with other aspects of these guidelines that address the importance of access to care, accommodation of a woman's personal support system, continuity of care, screening and assessment, and health promotion counseling.

Putting many of the elements of quality prenatal care into practice requires clinician time. However, fee-for-service models are disincentives to spending time with pregnant women. Moreover, in Canada the relatively few family physicians who offer maternity care combined with the low number of registered midwives puts pressure on obstetricians to provide care to both low- and high-risk pregnant women [52,53]. Collaborative models of care may be the answer not only to improving access to primary maternity care in Canada but also to enhancing the quality of prenatal care women receive [54]. Increasing access to midwifery care, which integrates many of the essential components of quality care because it is women-centred, embraces shared decision making, and incorporates emotional care [46], also may serve to promote quality prenatal care.

The findings of this study will be used to identify specific items for the Quality of Prenatal Care Questionnaire. Following the generation of a preliminary list of items, standard approaches to item reduction and validity and reliability testing will be used. If determined to have acceptable psychometric properties, the questionnaire can be used in future studies designed to explore different models of prenatal care and the ways in which they might enhance the quality of care women receive. Group prenatal care, as one alternative model, shows promise in delivering quality care and in influencing positive outcomes [7,33]. The Quality of Prenatal Care Questionnaire can be used in a variety of other studies. Research is needed to examine disparities in quality of care as studies have suggested it may vary for different populations of women. Wheatley and colleagues [14], for instance, found that low-income primiparous women had numerous negative experiences related to specific markers of patient-centredness: listened carefully, explained things, showed respect, and spent enough time. Studies of the ways in which quality of prenatal care varies by care provider also are warranted. Finally, research should examine the impact of quality care on a broad variety of maternal and infant outcomes and on future health service utilization. Preliminary evidence suggests the importance of specific dimensions of quality care, such as attention to lifestyle risk factors, adequate time with a care provider, and relationship-centredness, in improving maternal and infant health [4-7].

## Conclusions

Quality prenatal care is multidimensional and encompasses structure of care, clinical care processes, and interpersonal care processes. The study findings suggest the need to focus on more than the biomedical aspects of care and attend to elements of prenatal care that foster a meaningful relationship between a woman and her prenatal care provider. The promotion of quality prenatal care in clinical practice has implications for the training of current and future health professionals, the structure of care delivery, provider reimbursement schedules, and policy development. While optimizing the quality of prenatal care is not without its challenges, an investment in quality care has the potential to enhance the health of pregnant women and reduce the risk of adverse perinatal outcomes.

## Competing interests

The authors declare that they have no competing interests.

## Authors' contributions

WS and MIH wrote the grant application, directed the implementation of the study protocol, and had overall responsibility for the research. ST, PAJ, DY, DK, MEH, NAD, and EH contributed to the development of the study design and grant application. WS, MIH, ST, PAJ, and DY supervised participant recruitment and data collection in their respective settings. WS, SB, and MIH analyzed the qualitative data. WS and SB drafted the manuscript. All authors provided feedback on the draft manuscript, and read and approved the final manuscript prepared by WS.

## Pre-publication history

The pre-publication history for this paper can be accessed here:

http://www.biomedcentral.com/1471-2393/12/29/prepub
